# Guidelines for information about therapy experiments: a proposal on best practice for recording experimental data on cancer therapy

**DOI:** 10.1186/1756-0500-5-10

**Published:** 2012-01-06

**Authors:** Alejandra N González-Beltrán, May Y Yong, Gairin Dancey, Richard Begent

**Affiliations:** 1 Computational and Systems Medicine, University College London, Cruciform Building, Gower Street, London, UK; 2 Cancer Institute, University College London, Huntley Street, London, UK

## Abstract

**Background:**

Biology, biomedicine and healthcare have become data-driven enterprises, where scientists and clinicians need to generate, access, validate, interpret and integrate different kinds of experimental and patient-related data. Thus, recording and reporting of data in a systematic and unambiguous fashion is crucial to allow aggregation and re-use of data. This paper reviews the benefits of existing biomedical data standards and focuses on key elements to record experiments for therapy development. Specifically, we describe the experiments performed in molecular, cellular, animal and clinical models. We also provide an example set of elements for a therapy tested in a phase I clinical trial.

**Findings:**

We introduce the Guidelines for Information About Therapy Experiments (GIATE), a minimum information checklist creating a consistent framework to transparently report the purpose, methods and results of the therapeutic experiments. A discussion on the scope, design and structure of the guidelines is presented, together with a description of the intended audience. We also present complementary resources such as a classification scheme, and two alternative ways of creating GIATE information: an electronic lab notebook and a simple spreadsheet-based format. Finally, we use GIATE to record the details of the phase I clinical trial of CHT-25 for patients with refractory lymphomas. The benefits of using GIATE for this experiment are discussed.

**Conclusions:**

While data standards are being developed to facilitate data sharing and integration in various aspects of experimental medicine, such as genomics and clinical data, no previous work focused on therapy development. We propose a checklist for therapy experiments and demonstrate its use in the ^131^Iodine labeled CHT-25 chimeric antibody cancer therapy. As future work, we will expand the set of GIATE tools to continue to encourage its use by cancer researchers, and we will engineer an ontology to annotate GIATE elements and facilitate unambiguous interpretation and data integration.

## Background

### Recording experimental data

Recording and reporting experiments -- including their context, design, methods and results -- in an unambiguous manner is crucial for the advancement of biological and biomedical research. Systematic reporting enables data sharing and reuse, thereby avoiding repetition and inefficient use of resources. Unambiguous data recording allows for well-grounded comparisons and aggregation of experimental results. Analysis of the aggregated data as a large dataset is more likely to produce statistically significant results. It is also expected to support new hypothesis testing, simpler and better systematic reviews and meta-analyses. Moreover, the data could be used for teaching and training purposes [1]. In summary, the description of experiments should avoid different interpretations, and be presented in a way that allows for sharing and integration.

### Standardization initiatives for biological, biomedical and health research

The development and use of guidelines containing key information required to describe different kinds of biological and biomedical data are becoming widespread. For example, the practice of recording microarray data to the Minimum Information About a Microarray Experiment (MIAME) has been successfully adopted by the transcriptomics community. Many journals [2] and funders require the use of MIAME and it has been implemented in some microarray databases (such as ArrayExpress [3], the Gene Expression Omnibus (GEO) [4] and the Center for Information Biology gene EXpression (CIBEX) database [5]). *Minimum information *(MI) *checklists*, in general, advocate reporting transparency, better access to the data and support for effective quality assessment [6]. They have been shown to boost the value of the data produced in experiments and related publications, by encouraging more transparency and improving the access to the data and its quality assessment [6].

The Minimum Information for Biological and Biomedical Investigations (MIBBI) [7] project coordinates the development of these guidelines or checklists across the different biological sciences domains. In order to provide improved access to these minimum information checklists, MIBBI maintains a web-based portal with summary information, links and complementary information about them. The extra resources include data formats, controlled vocabularies, ontologies, tools and databases. Additionally, MIBBI coordinates the development and harmonization of the MI specifications. This coordination and harmonization process is important so that integration of data complying with different MI specifications is possible. Data integration is fundamental for secondary use of the data [6].

The EQUATOR [8] (Enhancing the QUAlity and Transparency Of health Research) network is an international initiative looking to improve the quality of reporting of clinical data for health research [9]. The network promotes transparency and accurate reporting by providing online resources and training for different stakeholders. These include developers of reporting guidelines, authors of research reports, journal editors and peer reviewers [9,10].

The existence of both the MIBBI and EQUATOR projects demonstrates a perception that checklists are beneficial to biomedical and health research. Some initial studies have aimed at determining whether the adoption of checklists proves beneficial [9]. Plint *et al*. [11] and Smidt *et al*. [12] have looked at the impact of journal support for checklists on the quality of publications (e.g. completeness and transparency), indicating better quality in both cases, but with room for further improvement. Plint *et al*. [11] present a systematic review of studies that either a) compared journals that adopted the CONSORT checklist against those that did not, b) compared CONSORT adopters before and after the checklist publication, or c) a combination of the previous two cases. On the other hand, Smidt *et al*. [12] analyse publications before and after thechecklist was published. Other study has shown the link between a Surgical Safety Checklist and the improvement of the death rate [13,14], by analysing the data before and after the introduction of the checklist.

Minimum information specifications or checklists [6,15], therefore, refer to the metadata, or 'data about the data', describing an experiment's context, design, methods and results. To ensure that this information is consistently reported, it is necessary to provide a unifying data format. In the case of microarray experiments, the Microarray Gene Expression Data (MGED) society first developed the MicroArray Gene Expression Mark-up Language (MAGE-ML) [16] to accompany the MIAME standard and enable the exchange of data between laboratories and public databases. However, MAGE-ML is too complex and not practical for laboratories without a dedicated bioinformatics support team. Thus, a subsequent development resulted in a simple spreadsheet-based format called MicroArray and Gene Expression TABular (MAGE-TAB) [17]. MAGE-TAB represents primary data and experimental metadata for microarray investigations using spreadsheets. MAGE-TAB is used by biologists for data collection, annotation, and exchange between tools and databases, including submissions to public repositories. Brazma [18] states that, despite the popularity of the MIAME checklist, the complementary MAGE-TAB format has not been as successful within the community, having a low rate of adoption.

To ensure that the data are interpreted in an unambiguous way, checklists and formats must be accompanied by controlled vocabularies or ontologies. A controlled vocabulary is a list of terms, each associated with a clear definition that makes it distinct and unambiguous. Maintenance of the vocabulary (i.e. additions, deletions, changes) is controlled. An ontology, on the other hand, is a formal representation (i.e. with a logical foundation) of the knowledge in a particular domain as a set of concepts and their relationships. An ontology provides greater interoperability than a controlled vocabulary. Continuing with the examples from the microarray community, the MGED society also produced an ontology based on the MIAME guidelines -- the MGED ontology [19,20] (MO) -- that defines unambiguous terms for the annotation of experiments; i.e. considering the elements specified in the checklist.

Thus, the three basic components of a reporting structure are [6,15]:

• Minimum information (MI) specifications or checklists

• Data formats: capturing MI in standard, non-proprietary formats

• Controlled vocabularies or ontologies: using unambiguous, standard terms

In summary, the checklists indicate *what should be reported*, the data standards specify *the format or syntax *to be used, and the terminologies or ontologies ensure that the meaning (or *semantics*) of the different elements is unambiguous.

### Recording therapy experiments

Therapy development involves activities ranging from target discovery, the design of a therapeutic agent, through to investigations of the effects of the agent in molecular, cellular, animal and clinical models. Consequently, therapy development involves interpreting and integrating information from heterogeneous domains.

While communities within the biological, biomedical and health research fields have developed guidelines to report various experimental data, none have specifically addressed therapy development. As in other areas of biomedical research [21], publications about therapy experiments often describe the data using free text or static tables in different formats, and might lack some of the information required to understand the experiment in detail.

A standard for therapy experiments would bring together different types of information, using, where possible, existing standards corresponding to the relevant sub-domains. The linkage of data from each sub-domain would produce a unified view of the different stages of therapy development. The immediate advantages of utilising a standard for this linkage are the avoidance of misinterpretations and repetition of time consuming tasks, as well as the minimisation of the risk to early phase clinical trials due to missing or misinterpreted data.

In this paper, we describe the Guidelines for Information About Therapy Experiments (GIATE) as a consistent information framework for linking diverse data types that can be applied to all the major types of therapy. Using GIATE, complex data sets can be linked to facilitate the understanding of the therapeutic system as a whole, contributing to optimise the efficiency and safety in the development of new treatments. One objective of the GIATE framework is to improve the communication between basic and clinical research by relating data from both ends of the development spectrum. Thus, with this translational medicine approach, we expect that more therapeutic insights may be derived from new scientific information.

GIATE originated from a collaboration among members of the Antibody Society [22], who worked at identifying the main elements that should be recorded for antibody therapy experiments [23]. Initially, GIATE was represented as a set of Common Data Elements (CDEs), as per the ISO/IEC 11179 metadata registries' standard [24]. The objective of this representation was to allow integration with the terminologies and data provided by the cancer Biomedical Informatics Grid^® ^(caBIG^®^) infrastructure [25,26], whose metadata registry is ISO/IEC 11179-based [27].

At a later stage, GIATE was extended to support other therapeutic approaches [28], while focusing on the importance and necessity of sharing data and data standards as required precursors of effective data sharing. Additionally, [29] showed how GIATE enables the establishment of a knowledge trail from molecular experiments to clinical trials, reflecting the steps of therapy development. This was exemplified with the key information elements for the Antibody-Directed Enzyme Prodrug Therapy (ADEPT) therapy, including information about the molecular target, therapeutic agents and experiments performed in molecular, cellular, animal and clinical models.

In this paper, we review and extend previous GIATE developments. The extension of previous work includes making GIATE objectives, scope, audience, design and structure explicit. As regards GIATE structure, we identify the modules that compose GIATE. We also introduce an extended GIATE checklist and spreadsheet-based data format. Finally, we consider a particular cancer therapy approach as a use case (CHT-25), and this is made available in GIATE structured spreadsheets.

## Results and discussion

### The GIATE reporting guidelines

GIATE (Guidelines for Information About Therapy Experiments) is a set of guidelines for the key information that should be reported about a therapy experiment, so that it can be properly understood, analysed and reproduced.

#### Objectives

The objectives of the development of GIATE are as follows:

• To provide a consistent information framework for reporting therapy experiments in a transparent way

• To support efficient data-sharing of therapy experiments' methods and results

• To guide the description of therapy experiments, enabling reproducibility, data reuse and re-purposing to avoid duplicated effort, support comparisons between experiments and increase the quality of the data

• To facilitate the integration of data coming from more than one experiment in a machine-processable way

• To allow for the possibility of obtaining aggregated and new knowledge coming from the statistical analysis of the data or mining of the aggregated information (data and knowledge mining)

• To develop tools to help the different GIATE stakeholders to use the GIATE guidelines

• To facilitate semantic publication of therapy experiments [30]

#### Scope

The scope of the GIATE guidelines is therapy experiments, such as small molecules targeting molecular pathways, engineered protein therapeutics, radio-immunotherapy, anti-vascular therapies, cellular therapies and others. For the design and development of new and improved therapies, it is crucial to be able to integrate laboratory and clinical data. Thus, GIATE aims at providing an information framework to record the properties of the target, the agent and models, the therapy investigation design, context and outcomes from the different studies.

#### Audience

These guidelines are mainly intended for use by *researchers *designing a therapy experiment, to assist them in collecting consistent data elements. Moreover, they are intended to act as a reference in order to form a coherent description of an experiment.

GIATE is also intended to be used by *journal editors *and *referees *to help confirm that certain data elements are uniformly presented in publications, and that their interpretation is unambiguous, allowing for the verification of the conclusions obtained.

Other potential beneficiaries of GIATE are the communities that regulate and fund therapy experiments. Within these communities, the Antibody Society [22] has adopted the GIATE specification.

Additionally, GIATE is intended to be used for educational or training purposes: we endorse the view that good research reporting habits should be introduced as early as possible in a researcher's career, to both students and young researchers [9]. The structure of GIATE presents the main properties of a therapy in a consistent manner, which facilitates the understanding of the therapy's strategy, intentions and achieved outcomes.

To benefit the different stakeholders, the GIATE project aims at providing tools that will help in making the extra effort of recording the experiments minimal and worthwhile.

#### Design

GIATE [31] is part of the MIBBI (Minimum Information for Biological and Biomedical Investigations) Consortium [6,32]. Following the MIBBI design guidelines used in other specifications [21,33], GIATE does not intend to provide an exhaustive list of data required or resulting from a therapy experiment. Instead, GIATE balances the trade-off between the depth of information required to record an experiment and the burden for the researcher to produce and maintain this metadata (or data about the data). Thus, the compromise between sufficiency and practicability [33] has been considered in GIATE's development.

GIATE is structured as a set of modules, most of which are specific to therapy experiments. Others, such as the citation module, are generic and could be reused within other guidelines. GIATE establishes the relationship between its constituent modules. Each module encapsulates the information related to a specific sub-domain of therapy development. A modular design guarantees that changes in a specific module will be local and will not affect the guidelines and tools related to other modules.

In comparison to guidelines that focus on specific assay results, such as MIAME [18,34] or the Minimum Information About Cellular Assay (MIACA) [35], GIATE can be considered a *'meta-guideline' *because it deals with high-level information from studies ranging from the lab to the clinic. These other guidelines form independent modules in GIATE, where appropriate. Users are therefore presented with domain specific guidelines if a higher level of detail is required to record any aspect of their data. GIATE, then, is the 'glue' linking diverse data to support translational research.

Another important feature of GIATE is that it is not static. As with other checklists such as the Minimum Information for Molecular Interaction experiments (MIMIx) [21], it is expected that through the increasing participation of the therapy community, GIATE will evolve to reflect the changing requirements in the context of a rapidly evolving therapeutics science. In this paper we present specific versions of the GIATE checklist and its related tools.

#### Structure

**Figure 1:**
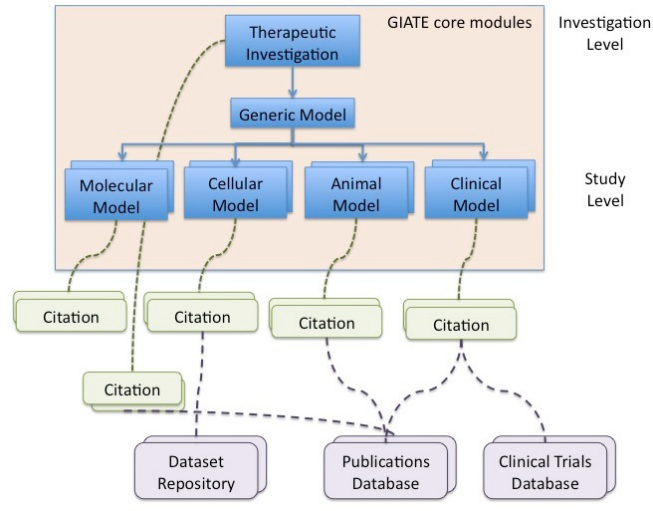
**GIATE modules This figure presents a schematic representation of the main modules of a therapy experiment: Therapeutic Investigation and the four Models in which the therapeutic can be applied (zero or more of each category including Molecular, Cellular, Animal and Clinical models)**. A supplementary model with metadata about citations is also depicted, as well as links to external databases.

Figure 1 depicts a schematic view of the main components of a therapy experiment, which are classified into the GIATE core modules, supplementary modules and links to external resources.

As per MIBBI guidelines, we consider the distinction between Investigation, Study, and Assay. An *investigation *refers to 'a self-contained unit of scientific enquiry' [6] that is characterised by an hypothesis or objective and a design, which is defined by the relationship between one or more *studies *and *assays*. Core GIATE modules can be seen as a tree, including the therapeutic investigation description at the root and more specific studies including data on the therapy development at the branches and leaves.

The main GIATE module is the *Therapeutic Investigation*, whose design is determined by the *Therapeutic Target *and the *Therapeutic Agent*. In turn, the *Agent *may be composed of one or more *Components*.

**Figure 2:**
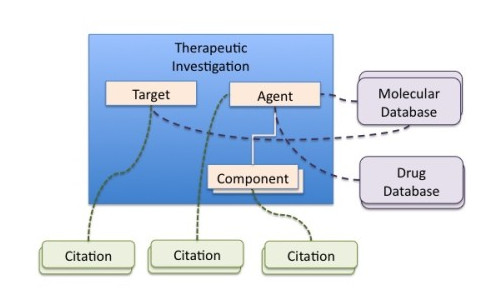
**GIATE Therapeutic Investigation This figure shows the main elements of a *Therapeutic Investigation*: the *Target *and the *Agent***. Moreover, the Agent may contain one or more components. Each of the elements might be associated with one or more *Citation *modules and linked to external databases.

Figure 2 presents the internal structure of the *Therapeutic Investigation *module, with sub-modules describing the target and the agent with its components. Supplementary modules for citations about the target, agent and components are also included, together with links to external resources.

**Figure 3:**
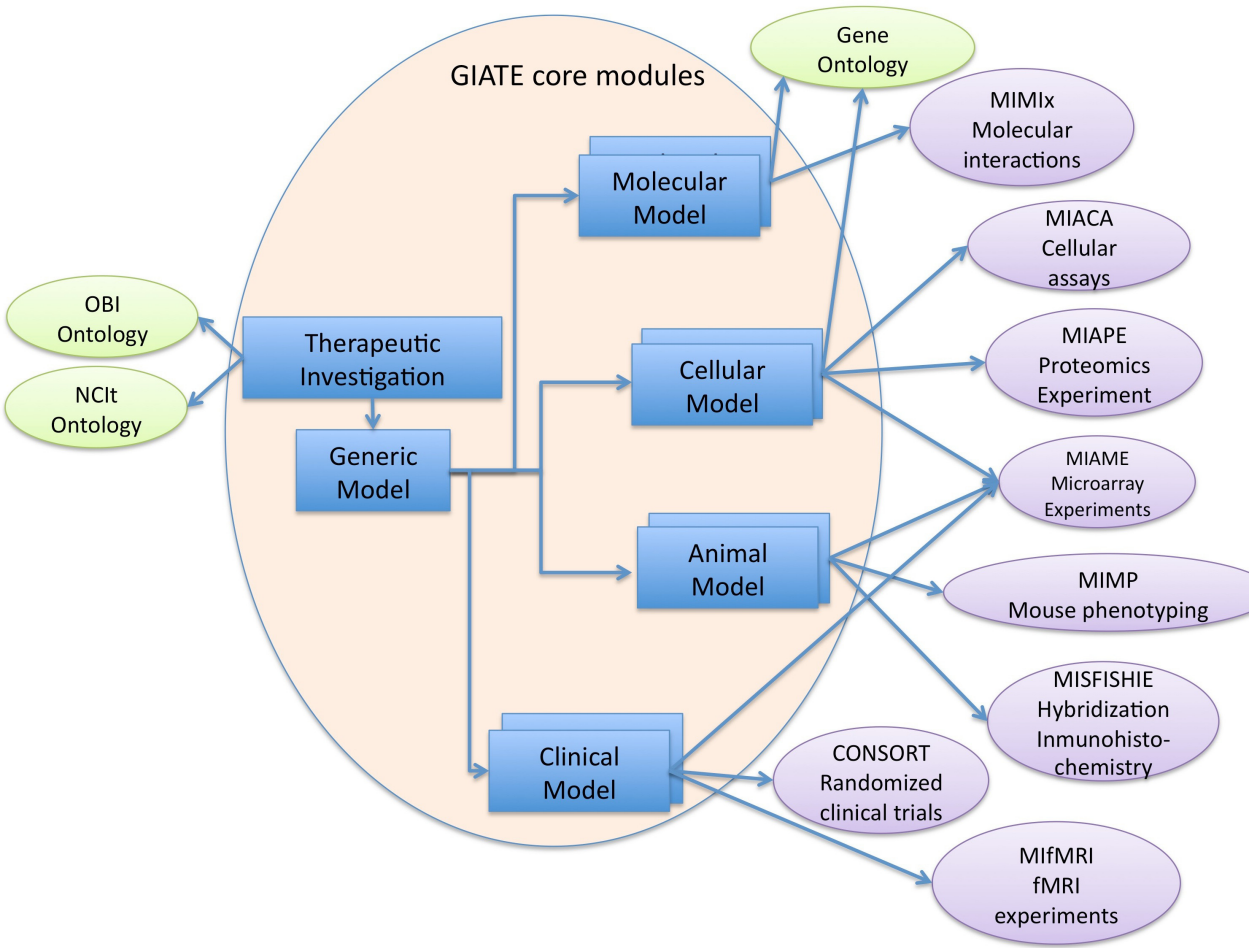
**GIATE and its relationship with other minimum information guidelines and ontologies 5**. This figure shows some of the ontologies and other minimum information guidelines that are relevant for each of the modules in GIATE. The relationships shown between each module and ontologies (in green) and MIBBI guidelines (in purple) are just presented as examples of potential ontologies/guidelines that might be considered when using GIATE.

The studies are represented by the different *Models *(see Figure 1) as each investigation may be applied to one or more models. Model types include: *Molecular, Cellular, Animal *(or pre-clinical) and *Clinical*. A particular *Investigation *may have been applied only to some of the models, for example to *Cellular *and *Molecular *models but not to the rest. Progress made from bench-side to bed-side can be tracked with the information in GIATE element types. Common characteristics of all the models are grouped into a generic *Model *module. Each of these models may have one or more assays. For example, a cellular model might contain information about cellular assays, which are reported to the MIACA checklist. When describing each of the modules, we discuss some of the relevant guidelines that researchers should consider for each sub-domain. Figure 3 shows some of the relevant guidelines per each module.

In addition to the GIATE core modules, we designed a module representing *Citations*, which is described in more detail when the GIATE checklist is introduced. In the future, other supplementary modules such as *Imaging *will be considered, given their role in therapy development [36].

#### GIATE Classification scheme

As described in the background section, GIATE was initially designed as a set of Common Data Elements (CDEs), as per the ISO/IEC 11179 standard for metadata registries [37]. A registry not only specifies the content that it maintains, but also the rules, operations and procedures that it uses to maintain its content. According to the registry standard, the set of CDEs determines a *classification scheme*, as they are grouped by the common characteristic of representing GIATE information.

**Figure 4:**
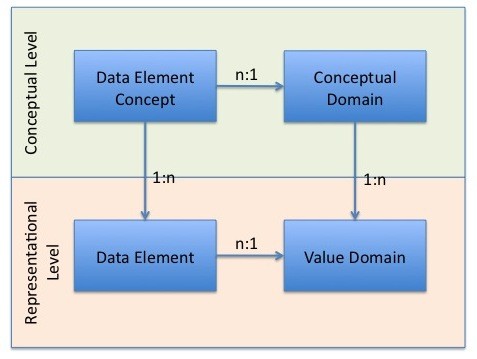
**GIATE ISO 11179 Metadata Registry Components This diagram is an overview model of the ISO/IEC 11179 metadata registry standard**. The figure is based on Figure 4 from [37].

Figure 4 shows a schematic view of the components of the ISO/IEC 11179 standard [37]. A data element is the basic container for data and it might represent an abstraction or an entity from some system. Data elements have both representational and semantic components [37]. In turn, the semantics involve two aspects: symbolic and contextual types. The contextual semantics comprise a data element concept, which indicates the types and characteristics of objects for which data are recorded [37]. The symbolic semantics come from a conceptual domain, which is a set of categories (enumerated or expressed with a description) representing the permissible or allowable values in a value domain. The representational level includes the data element itself as well as one or more associated value domains, specifying the set of permitted values [37]. We note that the metadata registry standard includes in a single model conceptual and representational aspects. The content to report is determined by a data element as an *ObjectClass*, a *Property *and a *Value Domain*.

Thus, a CDE involves simultaneously the three aspects of a reporting structure as seen before: what to record, how to record it, what is the meaning of the information recorded. Additionally, what to record (checklist) and how to do it (format) are intertwined between the conceptual and representational levels.

When identifying the sets of CDEs for a particular domain, it is recommended to reuse existing CDEs as much as possible, as this results in increasing interoperability of new data resources based on the new classification scheme with existing data resources. When developing the GIATE checklist we found that making this effort to reuse CDEs could impose constraints on the content of the key elements. For example, when dealing with the animal model some of the existing CDEs had an *ObjectClass *related to Animal while others had an *ObjectClass *related to Organism: *Animal Cancer Model Phenotype Description java.lang.String *and *Organism Species Name java.lang.String *[38]. However, when specifying *what to record*, having to use these two CDEs might be confusing, as in both cases we are referring to properties of the Organism used in the Animal Model.

In this paper, then, we present the key information elements independently of the CDEs, which can be associated a later stage. Thus, we divide GIATE in the three levels as determined by a reporting structure, and present the content to be reported independently of any data format.

#### GIATE Notebook

One of the tools developed to support GIATE is the GIATE Notebook-a piece of software that can be used as an electronic lab book to capture data on therapy experiments. The interface is composed of three panels: one for the GIATE elements, another one containing the CDE details showing associated terminology for each element and the third for data entry. The data produced with the GIATE Notebook can be exported as an eXtensible Markup Language (XML) document or in Portable Document Format (PDF).

More details on the GIATE notebook and its use for the ADEPT therapeutic investigation applied to an animal model [39] were presented in [28].

#### GIATE Checklist

We have developed a checklist with the key information that should be recorded about therapy experiments. The checklist main modules are as in Figure 1. In this paper, we will briefly describe each of the modules and will show the module concerning the clinical model in more detail, as this is the main component of the use case presented at section 2.3. The complete GIATE checklist file, version 0.1, is available as Additional file 1.

##### Therapeutic investigation module

This module involves some general information such as the goal and a brief description of the experiment, with an indication of the therapy type (e.g. antibody therapy), and a set of keywords and experimental factors. It also includes two sub-modules specifying the target and the agent, including possible components and their properties.

For the target, agent and components, it is required to specify their identifiers as available in public databases.

As in other minimum information specifications, such as MIMIx [21], we emphasise that ambiguous molecule identifiers, such as gene names, should be avoided. Instead, GIATE recommends that all molecules be identified by a database accession number from a public database (for example, the database resources of the National Center for Biotechnology Information [40]).

A database accession number identifies a unique molecule. In the case of a gene, giving its database accession number not only indicates its name but also the species from which the gene originated, which cannot be known by providing the gene name alone. It is noted that the annotations of proteins may change over time, for example when the coding sequence prediction programmes are updated [21]. These changes may invalidate the mapping of specific sequence positions such as those where binding domains are described [21]. Thus, as in MIMIx [21], an optional version number of the molecule or of the database is recommended in GIATE.

Table 1 presents the recommended public databases to identify each type of molecule. It is observed that therapeutic targets, agents or their components may not be present in public databases at the time of the experiment. In those cases, it is recommended to include as much information about the molecule as possible, such as its generic name, synonyms and references to publications describing it.

**Table 1:** Molecules Identification This table summarises the recommended public databases to be used for molecules identification

Molecules Identification	
Proteins	UniProt [41-43] or RefSeq [44,45]

Genes	Ensembl [46] or Entrez Gene [47,48]

Chemical Entities	PubChem [49] or ChEBI [50,51]

Drugs	DrugBank [52,53]

It is expected that the *Therapeutic Investigation *module will be included when reporting any kind of therapy experiment, regardless of which models are included.

##### Molecular model module

This module describes the experiments that study the strength of bonds between the target molecule and agent (or between components of the agent) as well as the distribution of the agent. Bond strengths are described in terms of affinity and avidity. Distribution is described in terms of concentration, volume and stability. This module also includes information about dose regimens.

A relevant minimum information specification that could be use to complement this module is MIMIx [21].

##### Cellular model module

This module describes studies at the cellular level. GIATE recommends describing the genetic and epigenetic profiles of the cell lines in terms of:

• The germline and somatic mutations

• Epigenetic silencing

• Gene expression fold changes.

This module is also used to record the distribution of both target and agent, in relation to specific dose regimens and the concentration and duration of drug exposure required for efficacy and toxicity.

##### Pre-Clinical (or Animal) model module

This module lists information elements relevant to therapy experiments in animal models. Some key elements are also present in the cellular model: e.g. genetic and epigenetic profile, and target distribution study. GIATE recommends recording details about the organism (its species name, phenotype description and developmental stage), as well as pharmacokinetics, pharmacodynamics and therapy outcomes in relation to different dose regimens.

##### Clinical model module

For a clinical model, GIATE recommends recording information such as the name of the trial, its phase, the number of patients, their medical conditions (associated, if possible, with an accession identifier from SNOMED CT or the Systematized NOmenclature of MEDicine-Clinical Terms [54]), the type of the trial (e.g. phase, single or multiple centre, open label, non-comparative dose escalation), its endpoints and objectives as well as eligibility and exclusion criteria used for participant selection.

Considering endpoints, i.e. measurements that can demonstrate the clinical benefit of the trial, some possible values are: overall survival (OS), time to tumour progression (TTP), objective overall response (ORR), complete response (CR) and time to treatment failure (TTF) [55].

Safety is a very important factor in the clinical model. In particular, for first-in-human trials, both the safe starting dose and higher dose levels or dose escalation criteria is paramount. Usually, the dose selection is based on specifically designed preclinical pharmacology and toxicology studies, ex vivo or in vitro experiments with human and animal cells and pharmacokinetics/pharmacodynamics (PK/PD) studies [56]. Thus, the dose selection is a clear example of how the previous modules in GIATE influence, and could be the source of the data, for the clinical model. However, the binding affinity of agent to target may differ across species and it is necessary to consider the relative potency between animal and humans [56]. There is evidence where life-threatening events directly related to the pharmacology of monoclonal antibodies were not predicted from pre-clinical toxicology studies (e.g. in the TGN1412 case) [56]. As a consequence, guidelines to explore the full pharmacological dose/concentration-response curve were introduced together with the concepts of Minimal Anticipated Biological Effect Level (MABEL) and No Observed Adverse Effects Level (NOAEL) [56]. Hence, GIATE recommends recording NOAEL and MABEL information.

As in the animal model, GIATE recommends to include the genetic and epigenetic profiles, target distribution, PK/PD studies, and therapy outcomes.

If applicable, GIATE recommends considering existing guidelines on health research for the clinical model.

For example, if the *clinical model *is a randomised controlled trial (RCT), the Consolidated Standards of Reporting Trials (CONSORT) should be used [57]. The CONSORT statement is part of the EQUATOR network presented in Section 1. CONSORT was developed by a group of scientists and editors aiming at improving the quality of reporting of RCTs, as overwhelming evidence showed that the quality of RCT reporting was not optimal [57]. The CONSORT statement consists of a checklist indicating the main information elements to include when reporting an RCT and a flow diagram [57].

##### Citation module

GIATE specifies a citation module. This is a supplementary module, as it is not specifically related to a therapy development. A citation is a reference to another entity.

When referring to bibliographic citations, the reference points to a publication such as a journal article, a book, a chapter, or a web page [58]. On the other hand, data citations consider reference to associated data.

Our *Citation *module is generic and allows to link any module or any of its elements to an entity, which can be a journal article, a database, a database record, a web page, a multimedia item, and so on. In Figure 1 we show how the *Therapeutic Investigation *or each of the *Models *can be linked to one or more citations.

### GIATE-TAB: a simple spreadsheet based format for cancer therapy experiment data

We have developed a simple spreadsheet-based format for recording information about GIATE: GIATE TABular (GIATE-TAB). The advantages of using a spreadsheet are two-fold: researchers are usually familiar with this format and it gives them some freedom on how they report the experiment. We believe that this is particularly important when the guidelines are in the first phases of development, as this will allow scientists to add information elements they consider relevant and feed back so that these can be incorporated in subsequent versions.

**Figure 5:**
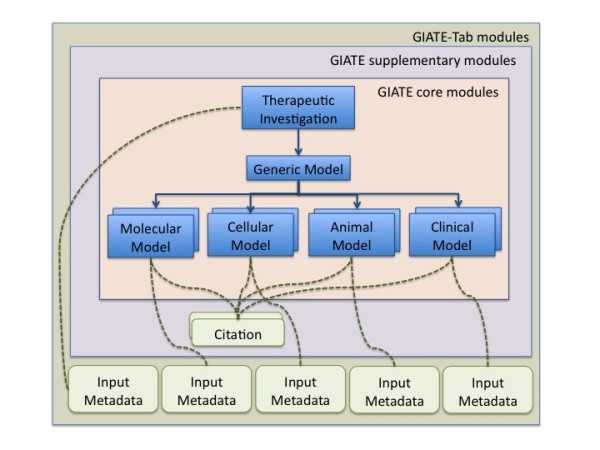
**Investigation Schematic view of GIATE-TAB, which apart from the information on GIATE guidelines includes provenance data at different levels of abstraction**.

GIATE-TAB includes not only metadata about the therapeutic investigation, as described in the GIATE checklist, but also some generic input metadata for each module (see Figure 5). This metadata is based on the Dublin Core (DC) [59] elements, and permits users to identify for each module: the title of the resource, a description, the creators, publishers and contributors to the metadata, the metadata source, date of creation, issuance and modification. These metadata elements are fundamental as they allow backtracking from the metadata to its sources. This is known as the provenance for each of the modules. The WC3 Incubator Group on Provenance define 'the sources of information, such as entities and processes, involved in producing or delivering an artifact' [60]. Their final report emphasises that the information about provenance of information is fundamental to establish if the data are to be trusted, to determine the way in which they can be integrated with other data and to support accreditation of the data originators in case of re-use [60].

The GIATE-TAB spreadsheet is provided as the Additional file 2.

In the near future, we expect to use tools such as the ISA Software Suite [61]. ISA stands for Investigation/Study/Assay and the ISA infrastructure [61] is a general-purpose format and freely available desktop software suite designed enabling curation of experimental metadata and supporting minimum information standards and, where available, submission to public data repositories. In particular, we will use the ISAconfigurator tool [61] to create a GIATE configuration, using the fields from the GIATE checklist. The GIATE configuration file will be used by biologists or cancer researchers to compile therapeutic investigation metadata using the ISAcreator tool [61].

### Use case: CHT-25 therapy

In this section, we present the use of the GIATE checklist and GIATE-TAB for a therapy experiment described in [62]. This experiment consisted of a Phase I trial of radio-immunotherapy with ^131^Iodine Chimeric Antibody (CHT25) to the IL-2 receptor in refractory lymphomas [62]. The main source of the GIATE metadata was the paper itself [62] and it was complemented with information available in the clinical trial protocol and data provided by the authors. The completed GIATE-TAB file is available as Additional file 3. As future work, we expect to link the GIATE-TAB information to the raw trial data. As CHT-25 is an ongoing study, we expect to re-use the recorded GIATE elements as the study progresses and show how data can be integrated to facilitate further comparison and analysis.

#### Therapeutic investigation module

**Figure 6:**
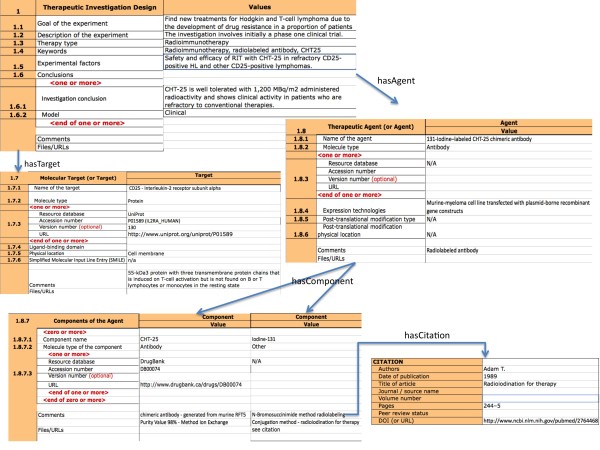
**GIATE-TAB for CHT-25, Therapeutic Investigation Section of GIATE-TAB for CHT-25 corresponding to the *Therapeutic Investigation***. The figure shows the elements of the investigation design for the CHT-25 therapy, the properties of the target molecule, the agent molecule and its two components. Moreover, we also show a citation module related to the radiolabeling method used for the ^131^Iodine component.

In the therapeutic investigation module (see Figure 6), we have included general information about the investigation: its goals, description, therapy type, experimental factors and its conclusions.

Additionally, the target (CD25, Interleukin-2 receptor subunit alpha), the agent (^131^Iodine labeled CHT-25 chimeric antibody) and its two components (CHT-25 and ^131^Iodine) are specified. CHT25 is a chimeric monoclonal antibody with murine variable regions and human constant regions. CHT25 was radiolabelled with ^131^I, an appropriate radionuclide for radio-immunotherapy as it has *β *emission length of 0.8 mm and γ emissions for imaging purposes.

The citation module is used to link to papers describing properties of the target [63,64] and the method used for the radioiodination of the antibody in the agent [65], as referred in the original paper.

#### Molecular module

The affinity of binding between CHT25 and IL-2 receptor is approximately that of IL-2 itself [62]. Previous results refer to the unlabeled antibody which has been used to prevent transplant rejection in renal patients. An alternative unlabeled antibody has shown short-term benefit in human T-cell lymphotrophic virus-associated lymphoma, where the IL-2 forms part of a growth pathway [62].

#### Cellular module

No cell lines studies exist for this therapy.

#### Animal module

There is no suitable representative animal model for ^131^I-CHT25. While Rhesus monkeys contain the same IL-2R epitope, they are not suitable for therapy studies. Toxicology for ^131^I-CHT25 has not been performed in pre-clinical models either [62].

Thus, within the reporting for the CHT-25 study the animal model includes comments about these facts.

#### Clinical module

The first section of the clinical model in GIATE-TAB includes general information about it:

• The objectives and endpoints of the CHT-25 study such as evaluation of the *toxicity, pharmacokinetics, immunogenicity *and *anti-tumour activity *of CHT25

• The number of patients, with description of the eligibility and exclusion criteria: the study involved 14 patients who had CD25 positive lymphomas (Hodgkin lymphoma, HTLV associated adult T-cell lymphoma and peripheral T-cell lymphoma) in which standard therapies had failed or were not tolerated [62].

• The study design or type, which in this case is single centre, open label, non-randomized, multiple dose escalation phase I trial.

• The conclusions: it was found that CHT25 has an important clinical activity in CD25 positive refractory lymphomas; it is relatively non-immunogenic with low toxicity at a non-myeloablative dose. Further studies are required to assess clinical effectiveness and these will be carried out in a Phase 2 trial.

This sub-module on *General Information *is linked to a citation module referring to the article [62].

For this therapy, information was included about individual patients. Elements referring to the type of lymphoma and treatment history are considered, including treatments such as chemotherapy, Autologous Stem Cell Transplant (ASCT), radiotherapy, time since last therapy, stage at therapy, and bone marrow involvement.

The sub-modules for *genetic/epigenetic profile, target distribution *and *pharmacodynamics *are not relevant for this particular investigation, and that is indicated in GIATE-TAB.

Information for the *dose regimen *is included for the investigation and for individual patients. The study consisted of a dose escalation using a standard dose of 10 mg of CHT25 antibody, with escalation of the radioactive iodine from 370*Mbq*/*m*^2 ^to 2960*Mbq*/*m*^2^.

CHT-25 was administered to 13 patients in 24 cycles. The dose limiting toxicity was determined at 2960 Mbq/m^2 ^with grade 4 myelosuppression in one patient. The patient failed stem cell re-engraftment and died of infection. The dose was reduced to obtain the maximum tolerated dose and 3 patients were treated at 1200 Mbq/m^2 ^with recruitment completing at 1480 Mbq/m^2^. Other toxicities were mild.

A *distribution study *was performed to analyse radioactivity uptake in target and non-target tissues. The main conclusions are summarised in GIATE-TAB.

Details of the *radiation dosimetry study *are also included. It is noted that this GIATE sub-module is only relevant for radioimmunotherapy experiments.

GIATE-TAB also includes information about *Pharmacokinetics (PK) studies*, i.e. how a drug or substance is absorbed, distributed, processed and eliminated in animals and humans. In order to study the PK for ^131^*I *in the CHT-25 investigation, blood samples were taken into EDTA blood tubes at the following time points, when possible: 1, 3, 6 and 24 h, then on day 2, 3, 6 and 9. The data presented in the paper [62] has been transcribed to GIATE-TAB including general parameters (e.g. median clearance for 50% and 90%) as well as per patient information. The latter comes from a tabular representation in the paper, giving the parameters of the PK interpolation curves per patient. The curve is either monoexponential, described by a single parameter, or biexponential, described by two parameters.

Finally, a sub-module indicating the *outcome *is also included, at the investigation and patient levels. Elements included are best response, Common Toxicity Adverse Events Grade and survival status. As the Cheson criteria [66] were used to classify the patient response (best response), e.g. as stable disease (SD), complete response (CR), partial response (PR) and so on. A citation module referring to Cheson *et al*. 's article [66] was linked to the *outcome study *sub-module.

#### Benefits of using GIATE for CHT-25

In this section, we indicate how GIATE has contributed to knowledge about CHT-25 and how having the data elements in the GIATE-TAB spreadsheet will help in understanding the different components of the therapy as well as facilitate secondary use of the data.

Firstly, the spreadsheet provides an **overall view of the CHT-25 therapeutic investigation**, which highlights the main points and their relationships. This process is simplified by the spreadsheet in comparison with the more time consuming task of reading the paper, the protocol, and if necessary, contacting the authors of the trial.

Secondly, the spreadsheet provides **links to external resources **that are available neither in the scientific article nor in the protocol. For example, the GIATE-TAB format for CHT-25 makes clear that the therapeutic target is CD25-Interleukin-2 receptor subunit alpha, accessible in UniProt (at http://www.uniprot.org/uniprot/P01589, version number 130). The specific link to UniProt allows users to uniquely identify the molecule that is mentioned in the paper. Thus, scientists wanting to analyse the CHT-25 trial could navigate to extra information about the CD25 molecule. Similarly, additional information about the agent component CHT-25 is accessible through the DrugBank database (at http://www.drugbank.ca/drugs/DB00074).

Thirdly, the paper provides static information presented in diagrams and tables. On the other hand, the GIATE-TAB for CHT-25 makes **re-use of the information in a dynamic fashion**. For example, while the pharmacokinetics analysis is available in the paper as Table 1[62], the figures cannot be immediately used to generate the interpolation curves. By having the data in GIATE-TAB, it is possible to generate these curves dynamically for further comparison and analysis of the pharmacokinetics data.

Finally, having the CHT-25 data in GIATE-TAB format facilitates **answering queries about the therapeutic investigation **much easier than having to go through the whole paper or protocol. For example, GIATE-TAB allows users to identify quickly what were the studies performed for the CHT-25 therapy and compare the Cheson score for the outcome of each patient at a glance. Additionally, GIATE-TAB is a step towards **answering queries about therapeutic investigations in a machine processable way**. As part of our future work, we intend to build a therapeutic investigations knowledge base, which will support to retrieve this kind of information.

## Conclusions

The development of therapy experiments involves activities ranging from target discovery to therapeutic design, and experiments to study the therapeutic approach performed in molecular, cellular, animal and clinical models. Interpreting this heterogeneous information in an unambiguous fashion is fundamental to draw new conclusions that interrelate data from the different models. GIATE has been presented as a set of guidelines divided into several modules, each dealing with one of the aspects or stages of the therapy development process. We have introduced the key elements of each of the modules and a use case for the CHT-25 therapy, focusing on collating GIATE information about the target, the agent and the molecular model and the phase I trial. As demonstrated in other areas of biological and biomedical research, producing a guideline to record experiments is the first step towards being able to report them transparently, compare them and integrate data coming from different experiments. We discussed the benefits of describing the CHT-25 therapy following GIATE.

As future work, we will develop an ontology associated with GIATE to facilitate both data annotation and data integration, by making the recorded elements unambiguous. Additionally, we will provide a machine-processable format to store information elements and facilitate automated data integration. This format will support building a knowledge base of therapeutic investigations with rich querying capabilities and links to other relevant data repositories.

We welcome feedback from the scientific community to help improve our proposal for recording therapy experiments. The GIATE project's email address is giate@ucl.ac.uk.

## List of abbreviations

caBIG^®^: cancer Biomedical Informatics Grid(R); ASCT: Autologous Stem Cell Transplant; CDE: Common Data Element; CiBEX: Center for Information Biology gene Expression; CONSORT: Consolidated Standards of Reporting Trials; CR: Complete Response; DC: Dublin Core; EQUATOR: Enhancing the QUAlisty and Transparency Of health Research; GEO: Gene Expression Omnibus; GIATE: Guidelines for Information About Therapy Experiments; GIATE-TAB: GIATE TABular; MABEL: Minimal Anticipated Biological Effect Level; MAGE-ML: MicroArray Gene Expression Mark-up Language; MAGE-TAB: MicroArray Gene Expression Tabular; MGED: Microarray Gene Expression Data; MI: Minimum Information; MIACA: Minimum Information About a Cellular Assay; MIAME: Minimum Information About a Microarray Experiment; MIBBI: Minimum Information for Biological and Biomedical Investigations; MIMIx: Minimum Information for Molecular Interaction; MO: MGED Ontology; NOAEL: No Observed Adverse Effects Level; ORR: Objective Overall Response; OS: Overall Survival; PD: Pharmacodynamics; PDF: Portable Document Format; PK: Pharmacokinetics; RCT: Randomised Controlled Trial; SNOMED CT: Systematized NOmenclature of MEDicine-Clinical Terms; TTF: Time to Treatment Failure; TTP: Time to Tumour Progression; XML: eXtensible Markup Language.

## Competing interests

The authors declare that they have no competing interests.

## Authors' contributions

RB, MY and AGB contributed to the GIATE checklist and presented it as a MIBBI checklist, extending the previous guidelines developed by RB, MY and others and initially expressed as a classification scheme for ISO/IEC 11179. MY had developed the GIATE notebook, including the initial set of guidelines. MY and AGB developed the GIATE-TAB format, based on the GIATE checklist. RB and GD reviewed the GIATE checklist and GIATE-TAB format. MY, AGB and GD completed GIATE-TAB for the CHT-25 use case. RB and GD verified that the information about the CHT-25 use case was correct. AGB wrote the initial version of the manuscript, which was revised and edited by the rest of the authors. RB provided vision, scope, and requirements analysis. All authors participated in revision and have read and approved the manuscript.

## Supplementary Material

Additional file 1 **The GIATE checklist version 0.1 is included as an additional file**. It is noted that the file contains links to terms belonging to different ontologies. Some of these terms belong to the NCI thesaurus and were extracted from the GIATE classification scheme. These terms are provided as a guideline but are not considered part of the GIATE checklist. The terms will be exploited during the development of the GIATE ontology. -- GIATE checklist version 0.1. Click here for file

Additional file 2 **The second additional file is the GIATE-TAB spreadsheet to be used in conjunction with the GIATE checklist, when compiling metadata about therapy experiments**. -- GIATE-TAB spreadsheet. Click here for file

Additional file 3 **The third additional file is the GIATE-TAB spreadsheet completed with metadata about the CHT-25 phase 1 trial**. -- GIATE-TAB spreadsheet for the CHT-25 cancer therapy. Click here for file
